# Hydrological simulation and uncertainty analysis using the improved TOPMODEL in the arid Manas River basin, China

**DOI:** 10.1038/s41598-017-18982-8

**Published:** 2018-01-11

**Authors:** Lianqing Xue, Fan Yang, Changbing Yang, Guanghui Wei, Wenqian Li, Xinlin He

**Affiliations:** 1 Hydrology and Water Resources College, Hohai University, Nanjing, 210098 P. R. China; 2 Shihezi University, Shihezi, 832003 P. R. China; 3 Jackson School of Geosciences, University of Texas at Austin, Austin, 78712 USA; 4 Tarim River Basin Administration, Korla, 841000 P. R. China; 5 Hohai University Wentian College, Maanshan, 243000 P. R. China

## Abstract

Understanding the mechanism of complicated hydrological processes is important for sustainable management of water resources in an arid area. This paper carried out the simulations of water movement for the Manas River Basin (MRB) using the improved semi-distributed Topographic hydrologic model (TOPMODEL) with a snowmelt model and topographic index algorithm. A new algorithm is proposed to calculate the curve of topographic index using internal tangent circle on a conical surface. Based on the traditional model, the improved indicator of temperature considered solar radiation is used to calculate the amount of snowmelt. The uncertainty of parameters for the TOPMODEL model was analyzed using the generalized likelihood uncertainty estimation (GLUE) method. The proposed model shows that the distribution of the topographic index is concentrated in high mountains, and the accuracy of runoff simulation has certain enhancement by considering radiation. Our results revealed that the performance of the improved TOPMODEL is acceptable and comparable to runoff simulation in the MRB. The uncertainty of the simulations resulted from the parameters and structures of model, climatic and anthropogenic factors. This study is expected to serve as a valuable complement for widely application of TOPMODEL and identify the mechanism of hydrological processes in arid area.

## Introduction

The deterioration of aquatic ecosystems due to changeable hydrological processes is an ongoing issue in many basins world widely^1–4^. Hydrological processes are affected by complex factors such as soil properties, land use type, climate, and topographic conditions and vary spatially and temporally^5–7^. Therefore, prediction of water resource availability is a difficult problem restricted by the implementation of water shortages and integrated river basin management in many basins^8,9^. Hydrological models are efficient tools to create new management strategies for better utilizing current hydrological theories^10–13^.

Numerous watershed hydrologic models have been adopted for the simulation of streamflow in the last decades to solve watershed problems^14^. These models include Soil and Water Assessment Tool (SWAT), and Hydrological Simulation Program Fortran (HSPF) and semi-distributed Topographic hydrologic model (TOPMODEL). Xie and Lian (2013) compared the performance of hydrologic simulation of HSPF and the SWAT model in the USA and found that HSPF can generate more accurate discharge predictions^15^. Although many models are powerful and efficient for solving watershed problems, they are difficult to calibrate when considering various hydrology and water quality processes, especially in the arid area^16,17^. Low vegetation coverage, thick aeration zone and unique climatic conditions contribute to the complicated hydrological processes in arid area. TOPMODEL is widely used all over the world^18^, but rarely consider the influence of topographic index and snowmelt on watershed runoff.

As an excellent representation of semi-distributed models, TOPMODEL is popular and widely used in watershed scales^19–21^. The semi-distributed Topographic hydrologic model (TOPMODEL) is a rainfall runoff model based on a simple theory of watershed hydrological similarity, with the topographic index as the index of hydrological similarity^22–24^. To improve the applicability of TOPMODEL in the Manas River, the internal tangent circle on a conical surface method proposed by Yong (2009) is used for computing topographic index distribution in this paper^24^. Furthermore, this paper includes the consideration of solar radiation and calculates the snowmelt using the improved temperature index coupled with TOPMODEL^25–28^. There are three general aspects contributing to uncertainty in hydrology: uncertainty in hydrological phenomena, model structures, and parameter estimation^29–32^. Beven (1992) proposed generalized likelihood uncertainty estimation (GLUE) method to estimate hydrological uncertainty, which represents the latest developments in this field^33,34^. Therefore, the uncertainty of model parameters is studied using the GLUE method.

The mechanism of water movement in an arid area seems complicated^35,36^. The Manas River Basin (MRB) has been experienced water scarcity, resulting in conflicts among water consumers at upstream and downstream regions, and degradation of its natural ecosystems^37^. The functions of river ecosystem has been significantly influenced by the climate change, irrational exploitation and utilization of water resources, such as irrigation and dam construction in the MRB^38^. The objective of this study is to simulate hydrological process using the improved TOPMODEL considering snowmelt and topographic index and identify the sensitive parameters for flow in the MRB, China. These results are expected to help us to further understand the mechanism of water movement in the arid area supplied from glaciers and snowmelt, generate more accurate discharge predictions and help planners to establish effective water utilization and allocation policies.

## Results

### Calculationz of topographical index

**Figure 1:**
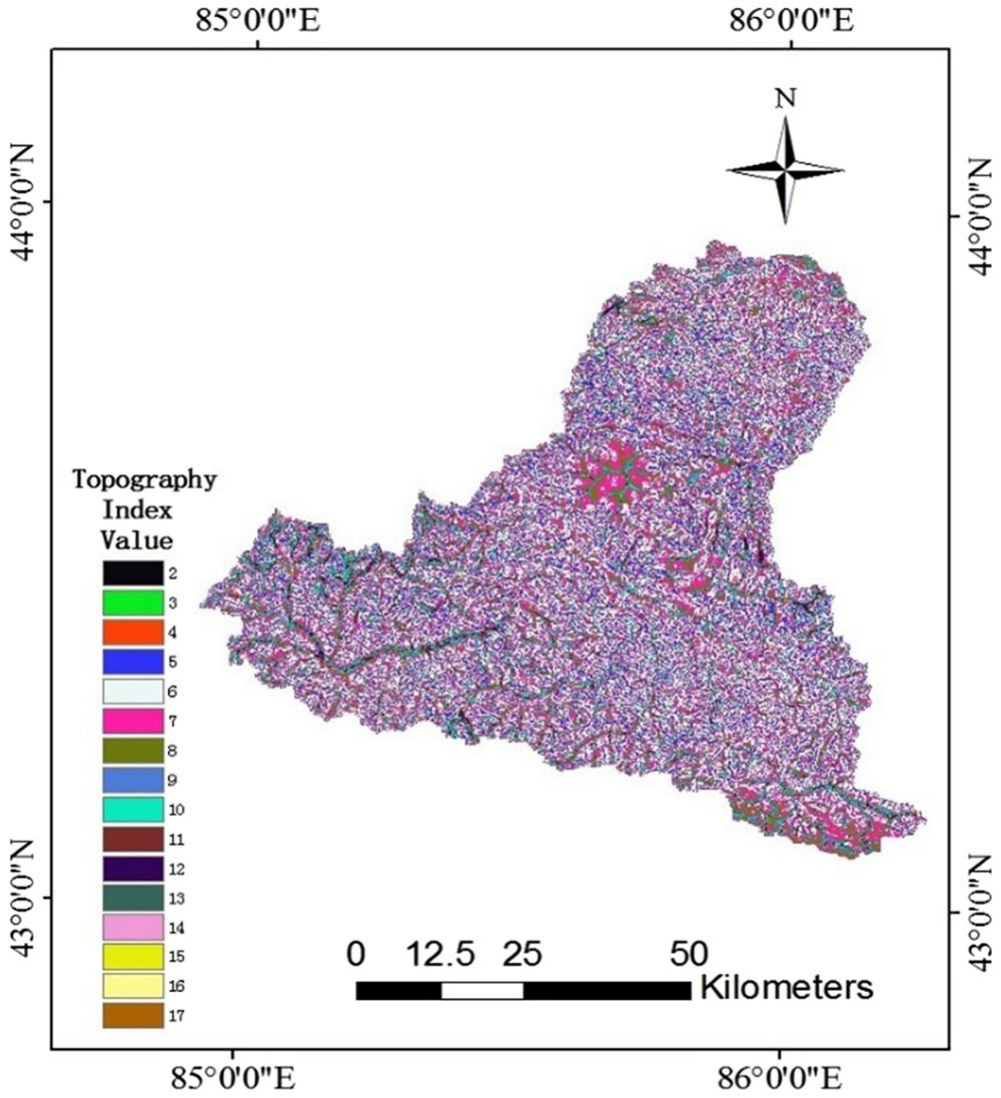
Distributed topographical indexes of the upper Manas River basin (Note: the map was generated using ESRI’s ArcGIS (version 10.1).

**Figure 2:**
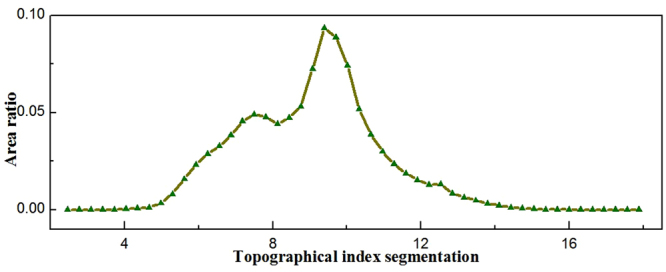
Plot of topographical index versus area ratio in the upper Manas River basin.

The single-flow direction algorithm is adopted for the calculation of the topographical index based on the digital elevation model (DEM). Since the grid points with similar topographical index have the same characteristic of hydrological response in TOPMODEL, the operating efficiency of the model will be improved in the region after the division of hydrological response units. It is not necessary to simulate the topographical index in all grids due to the similarity of the topographical index. The distribution function in the whole basin is generated by a statistical method with different topographical index values (Fig. 1). In this study, the region is segmented into 25 sections based on the topographical indexes, and the relationship of area ratios of topographical index is shown in Fig. 2. The minimum, maximum, mean, standard deviations of the topographical index are 2.9, 17.9, 10.3 and 4.5, respectively.

### Model Calibration and Validation

**Figure 3:**
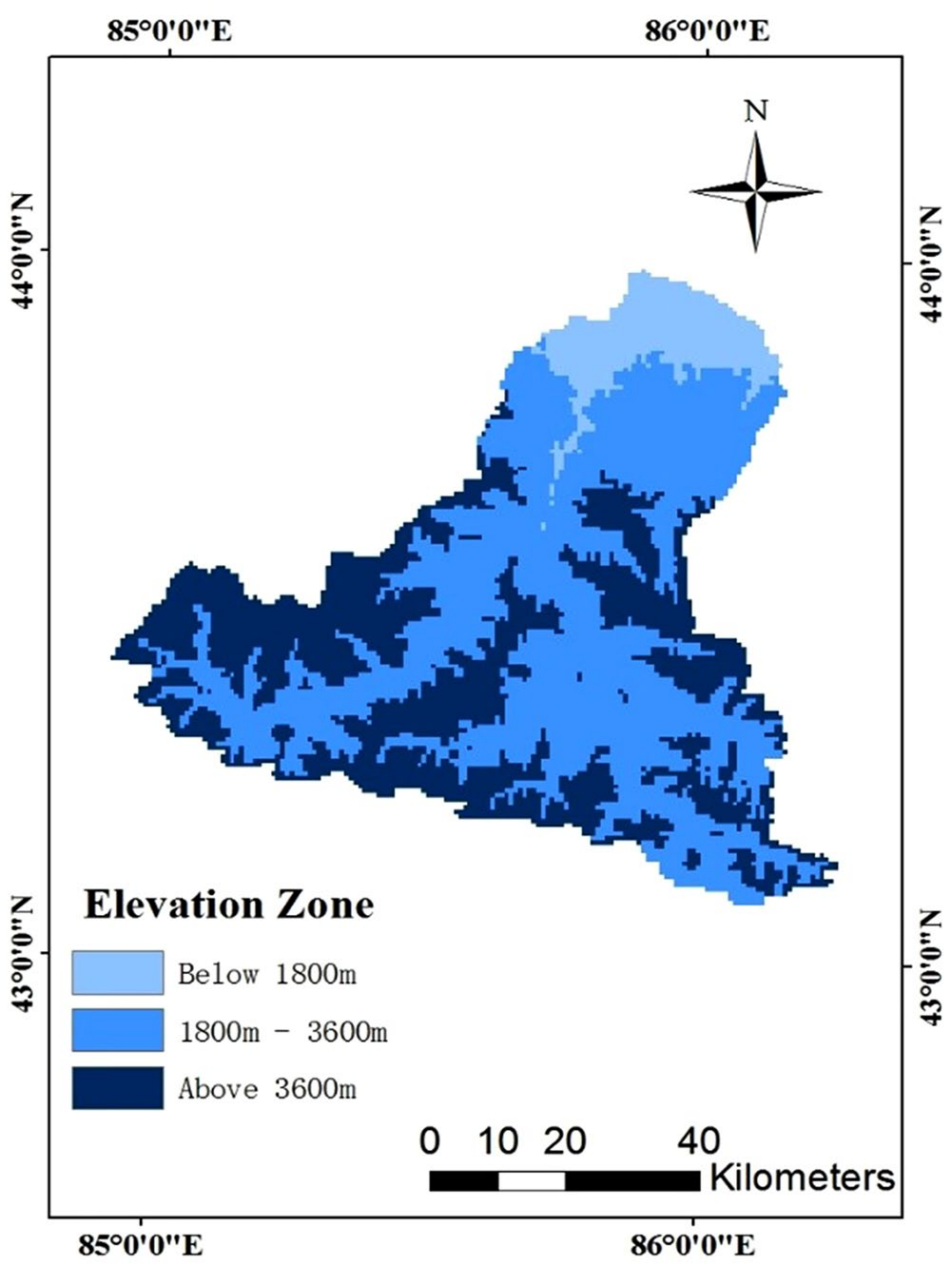
Vertical elevation of the mountain region in the Manas River Basin (Note: the map was generated with data available from the Chinese Geospatial Data Cloud using ESRI’s ArcGIS (version 10.1; http://www.gscloud.cn/).

The mountain region in the MRB illustrates vertical characteristics obviously. As the temperature decreases with rising elevation, the elevation has a great influence on snowmelt. To simplify the problem, we divide the mountain region vertically considering snow distribution and characteristics. The basin can be divided into three altitudinal belts. As shown in Table 1 and Fig. 3, regions with the altitude above 3600 m are defined as District C with high coverage of snow. Average ambient temperature in District C is below 0 °C. The region with altitude 1800~3600 m, district B, is the middle mountain area with patchy discontinuous coverage of snow. Regions below 1800 m are called district A.

**Table 1:** The three vertical distribution of the mountain region in the Manas River Basin.

Elevation(m)	Altitude zone	Area(km^2^)	Snow cover features
<1800	A	416.17	The instantaneous porphyritic discontinuous snow belt
1800~3600	B	2740.17	Patchy discontinuous snow belt
>3600	C	1956.27	Glacial and permanent snow

**Figure 4:**
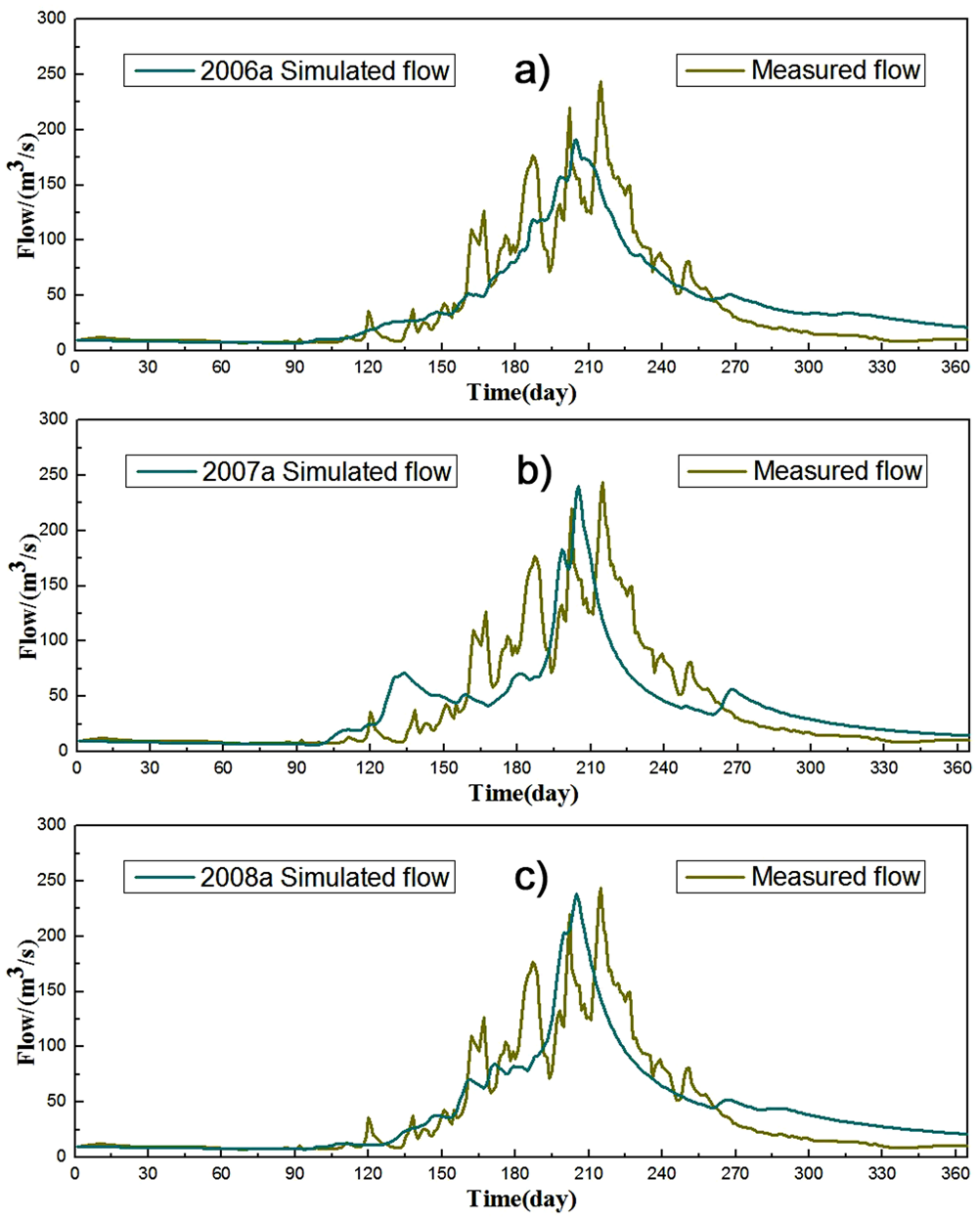
Daily runoff in the Manas River during the calibration period (during **a**) 2006 to **c**) 2008).

**Figure 5:**
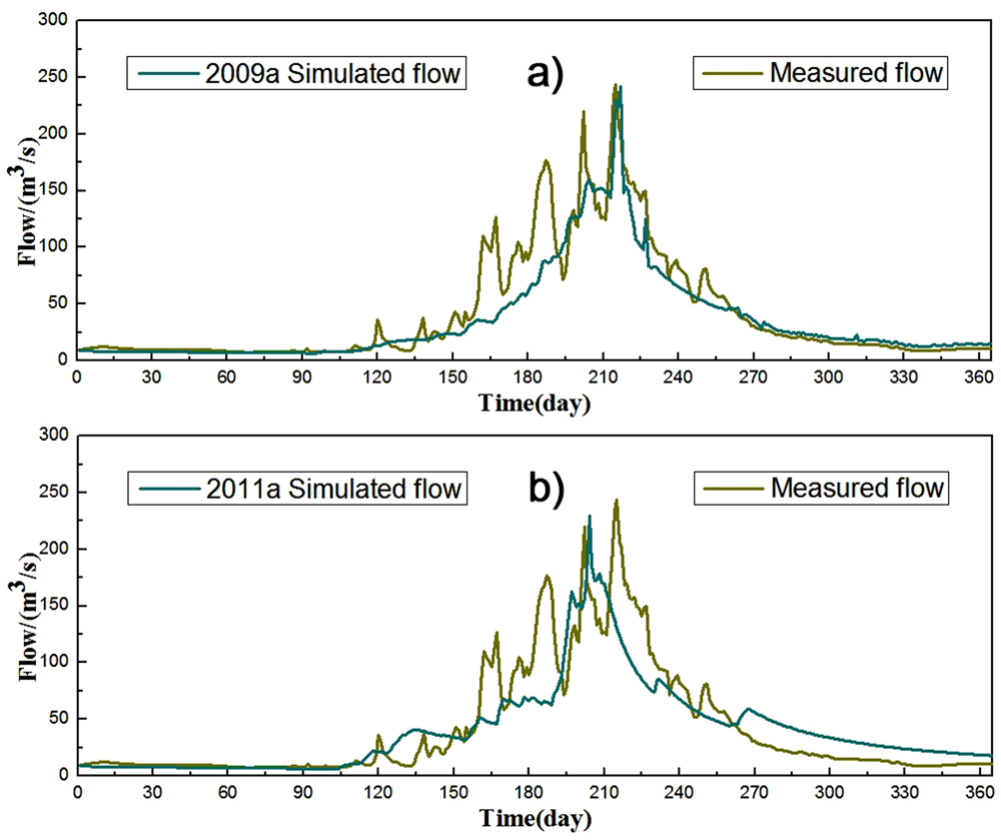
Daily runoff in the Manas River during the validation period (in **a**) 2009 and **b**) 2011).

The daily runoff data from 2006 to 2008 were chosen as the calibration periods, while 2009 and 2011 were determined as validation periods. Monte Carlo was used to generate model parameters randomly. The upper and lower limit for each sensitive parameter was obtained according to the historical data (Table 2). After setting the initial range, the parameters were calibrated using the variable domain decreasing algorithm. Figure 4 illustrates the simulated streamflow of the snowmelt-rainfall runoff model, the comparison of simulated and observed values and the general correlation between rainfall and runoff during the period of 2006 to 2008. After manual adjustment of the parameters, the coefficients of determination from 2006 to 2008 are 0.81, 0.61 and 0.75, respectively. Figure 5 shows the comparison of simulated and observed values and the general correlation between rainfall and runoff in the validation period. The coefficients of determination for the validation data (in 2009 and 2011) were 0.76 and 0.73, respectively. Therefore, the simulated result is significantly influenced by the accuracy of the snowmelt calculation. The results in the study reveal that the model performance is acceptable and comparable to runoff simulation.

**Table 2:** Parameters of the diurnal simulation.

Parameters	SR_max/_(m)	SR_0_/(m)	SZM/(m)	T_0_/(m^2^·h^−1^)	RV/(m·h^−1^)
Value	0.0301	0.0116	0.0464	3.4827	2329.194

Notes: SR_max_, SR_0_, SZM, T_0_, RV represent maximum water storage capacity in the root zone, initial saturated water shortage, exponential decay rate, effective infiltration rate, and effective overland flow runoff rate, respectively.

### Uncertainty Analysis of the Model Parameters

**Figure 6:**
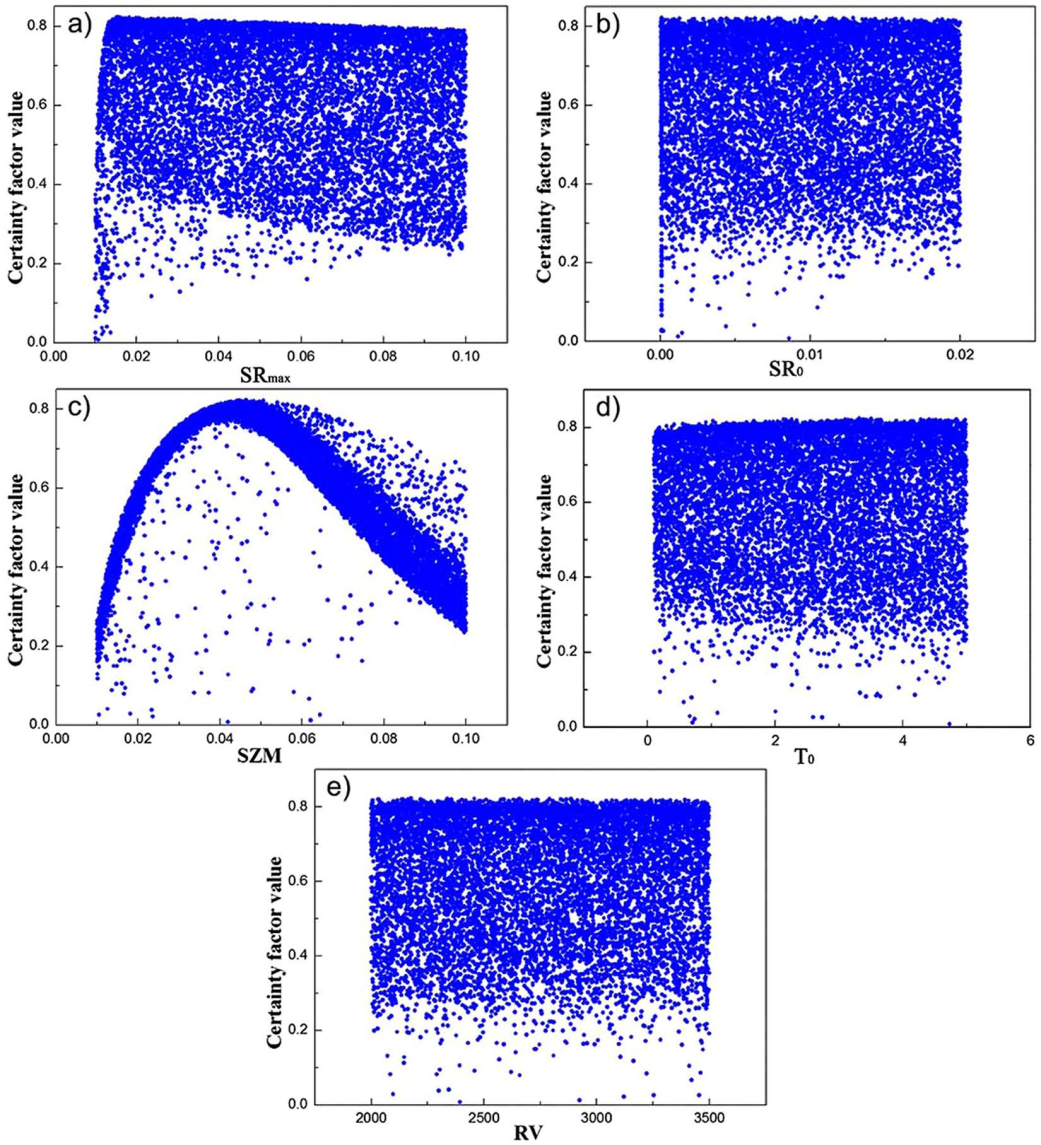
Dotty plots of parameters about (**a**)SR_max_, (**b**)SR_0_, (**c**)SZM, (**d**)T_0_, and (**e**)RV in the GLUE analysis(Notes: SR_max_, SR_0_, SZM, T_0_, RV represent maximum water storage capacity in the root zone, initial saturated water shortage, exponential decay rate, effective infiltration rate, and effective overland flow runoff rate, respectively).

The parameter sets with a sample size of 10,000 were generated through the Monte Carlo simulations based on the assumption of uniform distributions. The GLUE analysis was adopted to the simulation of daily runoff, and deterministic coefficient is chosen as the likelihood criterion. Table 3 shows the relationship between the parameters and likelihood value of the simulation of daily runoff. From the differences between certainty factor value and five parameters, it was found that four parameters (SR_max_, SR_0_, T_0_, and RV) are not sensitive for the runoff simulation. However, Fig. 6 shows that the exponential decay rate of SZM parameter has a significant impact on the simulation results. It indicated that the SZM is a sensitive parameter for the runoff simulation. Moreover, the value of SZM parameter is convergent to 0.0464 in the simulation finally.

**Table 3:** The value range of TOPMODEL parameters.

Parameter	Minimum	Maximum	Average
SR_max_	0.01	0.1	0.055
SR_0_	0	0.02	0.001
SZM	0.01	0.1	0.055
T_0_	0.1	5	2.553
RV	2000	3500	2750

Notes: SR_max_、SR_0_、SZM、T_0_、RV represent maximum water storage capacity in the root zone, initial saturated water shortage, exponential decay rate, effective infiltration rate, and effective overland flow runoff rate, respectively.

**Figure 7:**
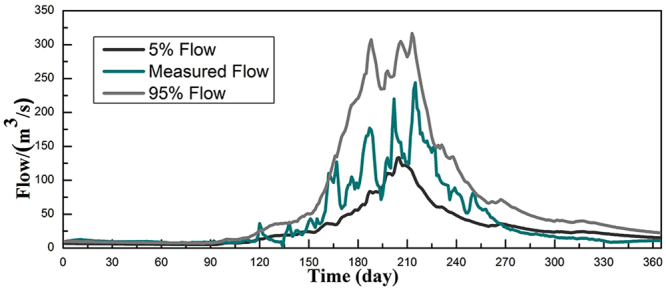
Uncertainty of the daily module in the Manas River Basin in 2006.

In this study, the simulations with likelihood value less than the threshold value of the parameters are considered to be zero, while the likelihood value larger than the threshold value of the parameters are normalized and sorted according to the simulated streamflow. Figure 7 shows the comparisons of the flood simulation results calculated with different conditions, including observations in 2006 and streamflow with the confidence interval of 95% and 5%. It indicated that the runoff simulation falls mostly within the 95% confidence interval except several peaks. However, the runoff simulation exceeds the 5% confidence interval almost three months in 2006.

## Discussion

In the current study, the topographical index was calculated using the geometric cone inscribed circle method. Figures 4 and 5 illustrate that the subsequent distribution of the topographical index is more intensive, with higher topographical index concentrated in the high mountain regions. It is signified that the regions with intensive distribution of the topographical index have high runoff accumulation trends. We employed an improved temperature indicator to calculate snowmelt, including influential factors such as surface radiation coefficient, glacier surface, sky permittivity, variation in solar radiation, and elevation. The calculated snowfall was input to TOPMODEL as the precipitation. After the several trials, the model application results illustrate that simulated result would be influenced by the precision of the snowmelt factor. Our results conclusively reveal that the model performance is acceptable and comparable to runoff simulation. However, accuracy of meteorological data may have a certain impact on the results of the model. Some errors may be related to the complex conditions of the climate and the underlying surface.

All the methods and data in this study are the respective influencing factors in the simulation. Evaporation data is obtained from the hydrological data at Ken Swart station. The improved degree-day method is adopted to calculate rainfall and snowmelt. Runoff is simulated by the topographical index curve and the geometric cone method. Moreover, the effect of parameters in TOPMODEL is also an important influencing factor to streamflow simulation. The selection range of the parameters is subjectively defined. In this study, the parameter debugging is manual. It implies that the parametric optimization may be not accurate and need for further study. All these factors may affect the simulation results and increase the processing uncertainty to the forecasting model.

Parameter equifinality is of great uncertainty to choose the so-called optimal parameter values. Parameter equifinality is resulted from the greater relevance of the model parameters, the defects of hydrological model structure and the complicated hydrological processes within the basin. In the current study, the parameter sets are randomly selected using Monte Carlo method. The sampling process could not be rigorous, otherwise performance period of the model will increase hugely. Therefore, shorter performance period allows the possibility of non-optimal sample sets, while reduces the accuracy of the simulation.

The impact of climate change and human activities has become great challenges for the simulation of streamflow^39^. The uneven distribution of rainfall, evaporation and temperature variation may affect the temporal and spatial characteristics of water resources. In fact, temperature and precipitation increased significantly and potential evapotranspiration showed a decreasing trend^37^. In this study, it can be focused on climate change effects on hydrological processes with snow accumulation (thickness) and snowmelt mechanism in the MRB. As for human activity, cultivated area expansion, agricultural irrigation and dam construction are the respective factors in the MRB^40,41^. Many researches revealed that more and more ecological water is consumed for the excessive expansion of irrigated area and agricultural water consumption is still climbing at an alarming rate despite the gradually improved water use efficiency and reformed irrigation management^38^. Meanwhile, reservoir construction has become great challenges for the sustainable development of water resources in the MRB. Therefore, it is unequivocally clear that the uncertainty from climate change and human activity may reduce the accuracy of runoff simulation consequently.

## Materials and Methods

### Study area

**Figure 8:**
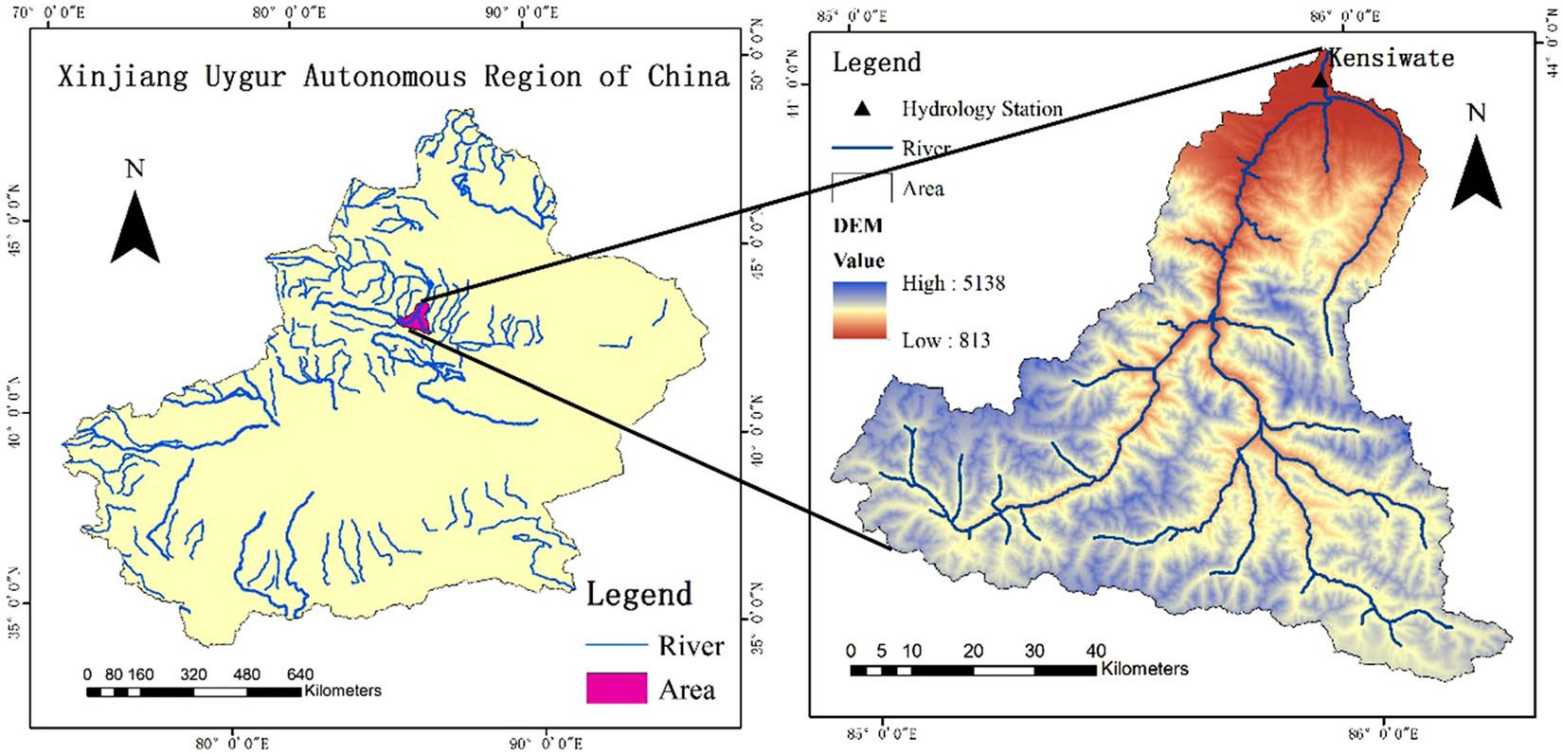
Sketch map of the Manas River basin and location of the hydrological stations (Note: the map was generated with data available from the Chinese Geospatial Data Cloud using ESRI’s ArcGIS (version 10.1; http://www.gscloud.cn/).

The MRB (85°01′~86°32′E, 43°27′~45°21′N) is located at the northern foot of the Tianshan Mountains in Xinjiang, and along the southern margin of the Junggar Basin (Fig. 8). The total area of the MRB is 1.98 × 10^4^ km^2^, while the plain area is 1.46 km^2^. The region has middle temperate continental arid climate, with a vertical climate change characteristic. The streamflow mainly comes from snowmelt and precipitation^42^. The precipitation distribution is uneven due to the influence of water vapor sources, topography, and latitude. Snowmelt water in Tianshan Mountain accounts for 47% of the annual volume of streamflow in the MRB. The streamflow generates in the mountain area, and dissipates in the plain area^43^. The Ken Swart hydrological station (43°58′,85°57′E) was started in 1955, and measured various hydrological parameters for the Manas river region. The MRB is a typical comb-like river system, with runoff distribution in the mountains above the mountain-pass, and loss below the mountain-pass.

### Data

Annual hydrological and meteorological data were collected from the Ken Swate hydrologic station, including precipitation, temperature, evaporation, streamflow, and so on. The simulation period was from 1th January, 2006 to 31th December, 2011. However, the data in 2010 is not complete, the series of streamflow excludes the data in 2010. Since the catchment area of the upper MRB is not large, the precipitation, evapotranspiration, runoff, and temperature data from the Ken Swart hydrologic station were used to represent the whole region. The calibration period was 1th January, 2006 to 31th December, 2008, and the validation period was 1th January, 2009 to 31th December, 2011 (excluding 2010).

Land use and vegetation data were collected from resources and environment science data center in Chinese academy of sciences (http://www.resdc.cn). Land use and vegetation cover changes cause consequential changes in local energy and water balance, and have effects on surface runoff, including the kinetic and potential energy, and the time and space distribution of the runoff. The difference of climate, underlying surface condition, the vegetation types and the rainfall intensity also have a profound influence on regional hydrological processes^20,44^. Therefore, the mechanism of runoff generation is studied under the condition of different land types. Seen from Table 4, this study classified the land types using the land resource classification method proposed by Chinese Academy of Sciences (CAS).

**Table 4:** Land use classification.

NO.	Name	Remarks
1	Cultivated	Herbaceous species, Aquatic plants
2	Forest	Woodland, shrub forest
3	Grassland	Growing herbaceous plants
4	Water	Natural land and water facilities
5	Urban and buildings	Urban and rural residential and other uses, such as mining, transportation.

The classification of Table 4 is not the only possible option. To meet the objectives of a given study, classification is often combined with the land vegetation cover types and region, which allows deeper segmentation^27^.

### Methods

#### The hydrologic model

**Figure 9:**
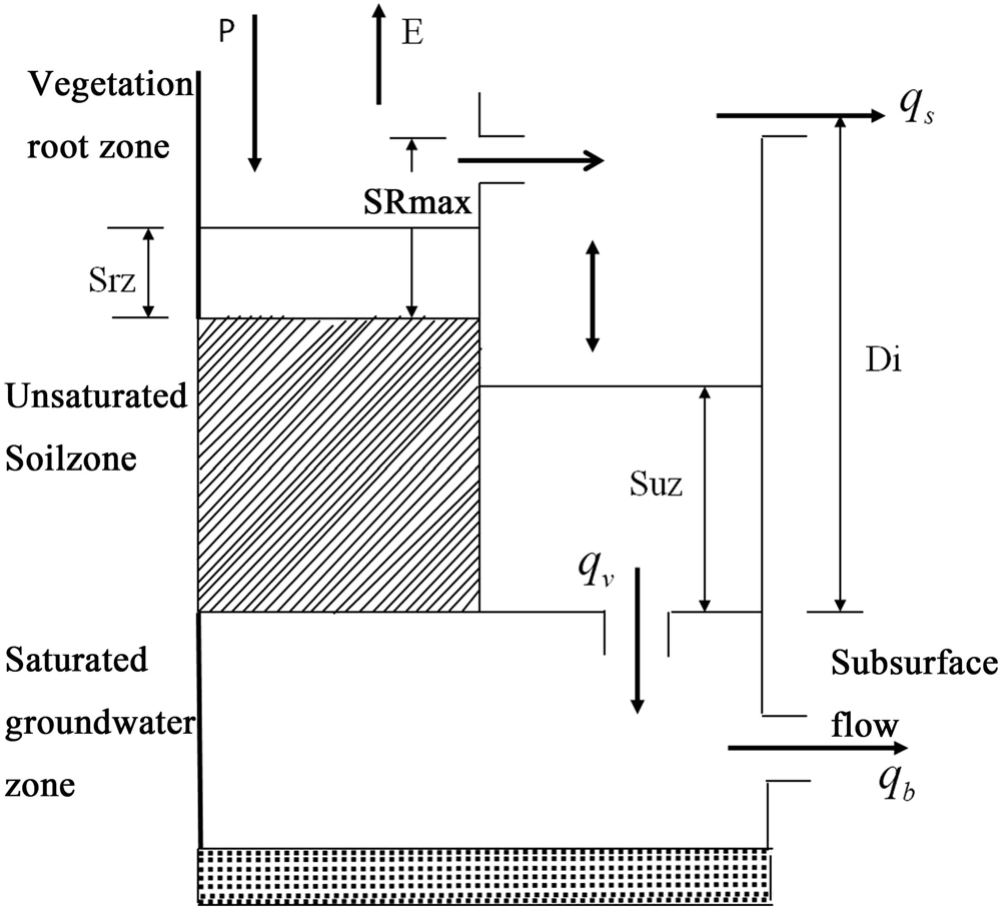
Diagram of TOPMODEL unit grid of soil moisture movement (Note: *P* represents precipitation, *E* represents the evaporation, ${S}_{rz}$_,_ ${S}_{uz}$ represent vegetation root zone, unsaturated zone, respectively. ${q}_{v}$, ${q}_{b}$, ${q}_{s}$ are infiltration, base flow and saturated slope flow. SR_max_ represents maximum water storage capacity in the root zone).

TOPMODEL is a physically based semi-distributed model for streamflow simulation, and has been widely used since 1970^23,45^. In this study, the TOPMODEL is used as a reference tool for simulating hydrological process in the MRB. In this hydrological model, the basin topography index is used to describe and explain the trend and movement of streamflow along the slope caused by gravity drainage. The soil of any place in the basin is divided into three different water-bearing zones: vegetation root zone (${S}_{rz}$), unsaturated zone (${S}_{uz}$), saturated zone (${Z}_{i}$).The basin was divided into grids by the digital elevation model. The hydrokinetic process for each cell is shown in Fig. 9. After the precipitation (*P*) satisfied the interception of plant canopy and filled the hollow, it infiltrates into the plant root zone and supplies the water shortage first. Some of the moisture stored in this zone evaporates while the remainder (${q}_{v}$) moves into the unsaturated soil zone, infiltrating vertically at some speed. In the saturated underground water zone, moisture moves laterally and forms a subsurface flow (${q}_{b}$(also called base flow)). The surface of saturated underground water varies with time due to the moisture infiltration and subsurface flow. If the level of underground water rises to the surface of ground, it forms a saturated surface, and produces a saturated slope flow (${q}_{s}$). Runoff occurs in saturated surface areas or source areas. The total moisture displacements, ${Q}_{b}$ and $\,{Q}_{s}$, can be obtained according to the formulas (2) and (3). Thus, in TOPMODEL, the total runoff ($Q$), is the sum of subsurface and slope flows,

1 $$Q={Q}_{b}+{Q}_{s}$$

2 $${Q}_{s}=\frac{1}{{\rm{\Delta }}t}\sum _{i}max([{S}_{uz,i}-max[{Z}_{i},0]],0){A}_{i}$$

3 $${Q}_{b}={Q}_{0}\cdot {e}^{-\bar{Z}/{S}_{zm}}$$

3 $${Q}_{0}=A{T}_{0}\cdot {e}^{-{\lambda }^{\ast }}$$

4 $${{\rm{\lambda }}}^{\ast }=\frac{1}{A}{\int }_{0}^{A}\,\mathrm{ln}(\frac{{{\rm{\alpha }}}_{i}}{{\tan {\rm{\beta }}}_{i}}){\rm{d}}A$$

where ${A}_{i}$ can be obtained by the curve between topographical index and area ratio in the upper Manas River basin (Fig. 2), ${\rm{\Delta }}t$ is the time step, λ is the topography index, ${S}_{zm}$ is the maximum of the depth of water storage in unsaturated areas,*T*_0_ is the effective infiltration rate. Normally, the time step is one hour in TOPMODEL. However, considering the observational data in the mountain regions, we used a time step of one day. There were 5 sensitive parameters among the 12 available parameters: the root zone maximum water storage capacity (SR_max_), initial saturated water shortage (SR_0_), exponential decay rate (SZM), effective infiltration rate (T_0_), and effective overland flow runoff rate (RV).

#### Topographical index

Topography is an important factor for precipitation, runoff and hydrological processes in land surface^22^. It is also the dominant factor in the spatial distribution of temperature, precipitation, soil, and vegetation within the basin, reflecting the controlling effect of gravity on the movement of water. The topography index assumes that topography drives flows. It generally correctly predicts the accumulation of flow in topological low spots or areas where the flows accumulate. The topography index is defined as:

5 $${\rm{\lambda }}=\,\mathrm{ln}(\frac{{\rm{\alpha }}}{\tan \,{\rm{\beta }}})$$

where λ is the topography index, α is the upslope contributing area per unit length of contour, and β is the topographic slope of the cell.

**Figure 10:**
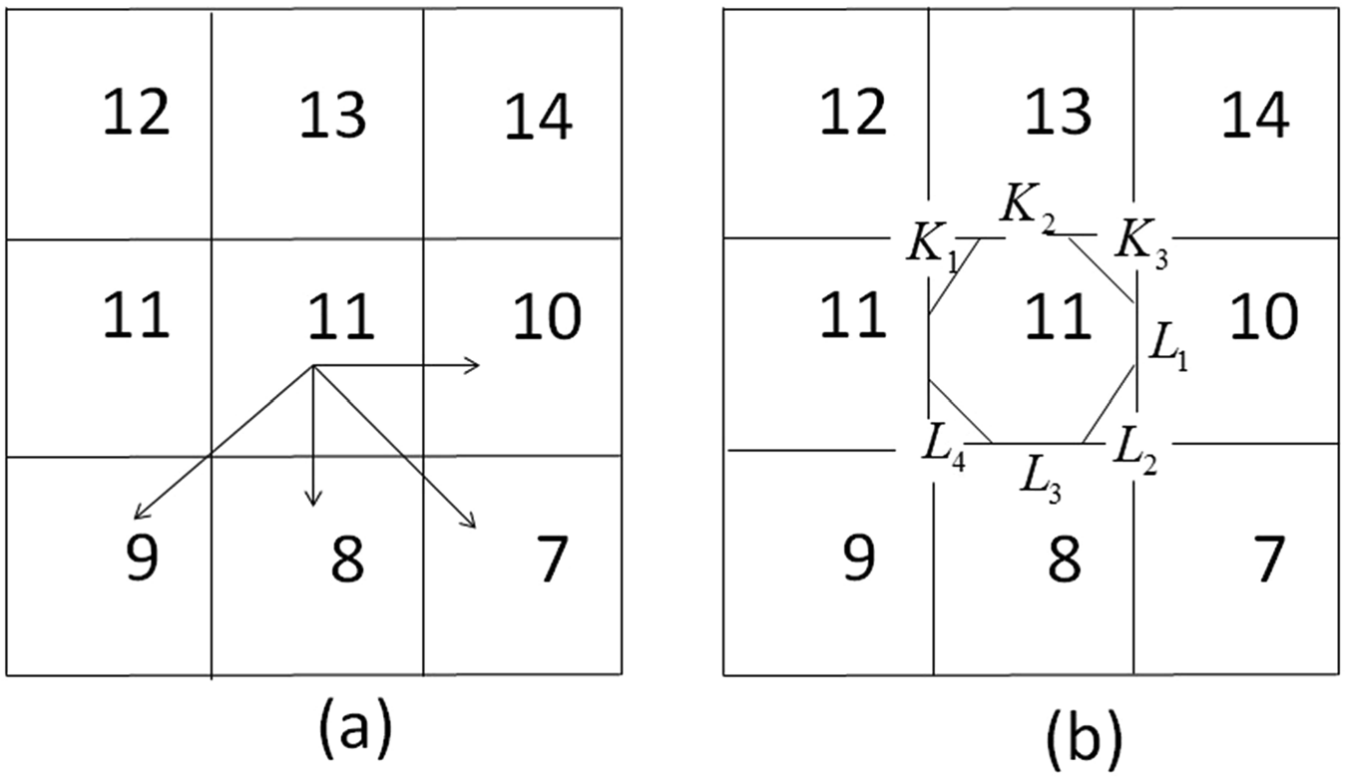
Multi direction flow distribution method.

**Figure 11:**
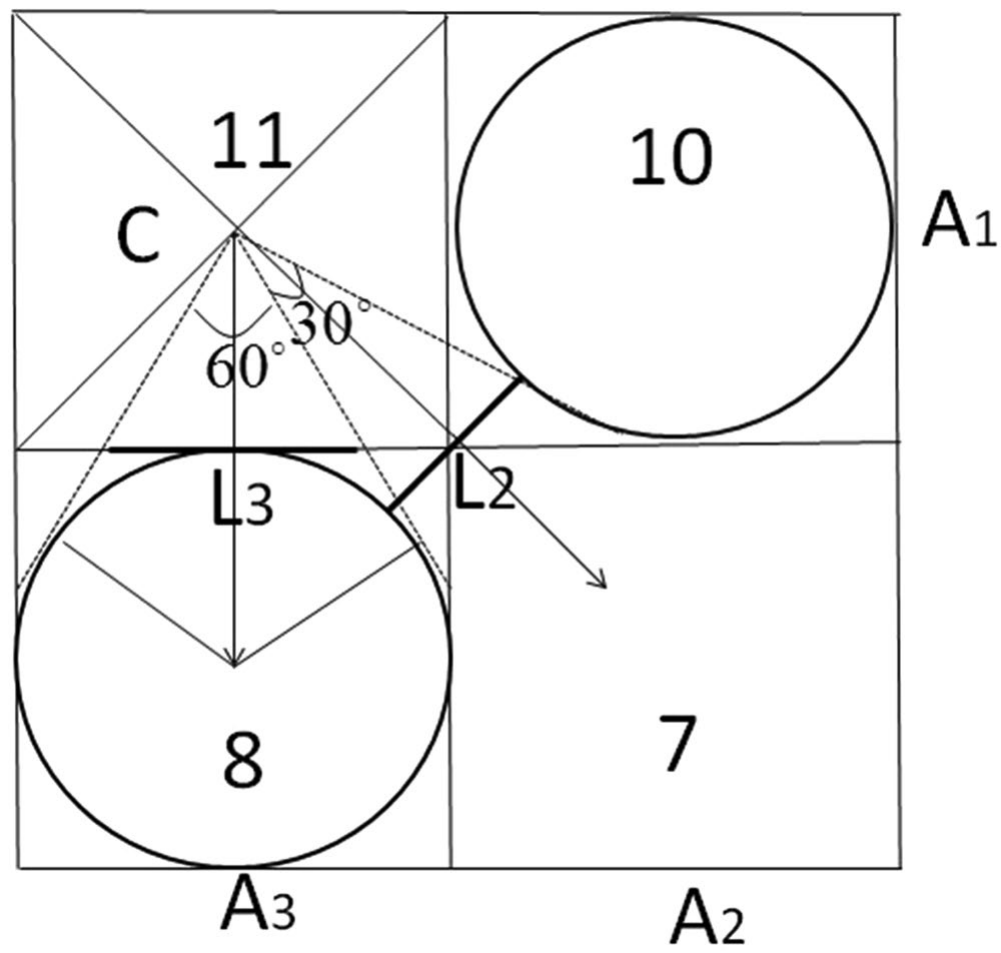
Geometric cone inscribed circle method of calculating the length of effective contour.

Suppose *A* is the total catchment area above one point and *L* is the width of a contour perpendicular to the direction of flow, then α = A/L. The slope of local surface ($\tan \,{\rm{\beta }}$) can be calculated from direction. The first step is to analyze the flow direction, then estimate A and L in the direction. Since geometric inscribed circle method is more accurate to reflect the hydrological similarity of real catchments, the geometric inscribed circle method is adopted in this study and the calculation principle is as follows:

Suppose four grids are extracted, as shown at the bottom right corner in Fig. 10. Seen from Fig. 11, point C is the central point of the calculation grid (11). The two grids 8 and 10 are slope downwards toward point C. We form tangents of circles A1 and A3, as shown in Fig. 11. Streamflow gathered from upslope areas moves into grid 11 and focuses on point C. Streamflow in grid 11 uniformly flows into grids 10, 8, and 7 along the downhill of conical surface. Runoff travels along the effective contour L3 and passes within the two tangents of A3, then flows into grid 8. This runoff is cumulative flow distributed from the computational grid. Other runoff goes through L2 and flows into grid 7. This part of the runoff flows into the grid in diagonal direction along the downhill grid in the main direction.

#### Snowmelt Module

Air temperature is the single index in the traditional snowmelt model following the energy balance model^28^. To ensure well performance for streamflow simulation, the improved calculation method of snowmelt is adopted to the snowmelt model. This method considers factors such as solar radiation and topographic features which are ignored in the traditional method. Solar radiation can be estimated without being imported by meteorological data, and the topographical factors (such as slope and slope direction) reflect the differences of spatial units. The time period is one day consistent with the usual observation of the snowmelt rate. The model is defined as:

6 $$ME=(MF+{a}_{\frac{snow}{ice}}I)(TP-TB)$$

where MF is the snowmelt factor (mm/°C · d), ${a}_{\frac{snow}{ice}}$ is the correction coefficient of sun radiation on the snow or glacier surface, *I* is the potential solar radiation in the surface (${\rm{W}}/{m}^{2}$), and *TP* and *TB* are the average ambient ice surface temperatures (° C), respectively. $MF$ and ${a}_{\frac{snow}{ice}}$ are set from prior experience, while *I* is related to the level of solar radiation above atmosphere,

7 $$I={I}_{0}{(\frac{{R}_{m}}{R})}^{2}{{\phi }_{a}}^{(\frac{P}{{P}_{0\cos Z}})}\,\cos \,\theta $$

where *I*_0_ is the solar constant($1368{\rm{W}}/{m}^{2}$),$\frac{{R}_{m}}{R}$ is the coefficient of eccentricity in the Earth’s orbit, where *R*_m_ is the average distance between the sun and earth, and *R* is the instantaneous distance between the sun and earth, ${\phi }_{a}$ is the average atmospheric transmittance in clear sky, and *P*, *P*_0_ and *Z* are explained below.

8 $$\begin{array}{c}\frac{{R}_{m}}{R}=1.00011+0.034221\,\cos \,{\rm{\Gamma }}+0.00128\,\sin \,{\rm{\Gamma }}+0.000719\,\cos \,2{\rm{\Gamma }}\\ \quad \,\,\,\,\,+0.000077\,\sin \,2{\rm{\Gamma }}\end{array}$$

where ${\rm{\Gamma }}$ is defined as,

9 $${\rm{\Gamma }}=2{\rm{\pi }}(\frac{i-1}{{n}_{a}})$$

The transmittance in clear sky is

10 $${\phi }_{a}=A+B\,\cos (2{\rm{\pi }}(\frac{i-{i}_{f}}{{n}_{a}}))$$

where *A* is empirical constant (0.64) in $\,{\phi }_{a}$; *B* is emission factor (0.12); $i$ is day number of the year; *i*_*f*_ is the day number factor in a year, in the southern hemisphere *i*_*f*_  = 174, whereas in the northern hemisphere *i*_*f*_  = 0; and *n*_*a*_ is the number of days in a year. The atmospheric pressure is

11 $${\rm{P}}={P}_{0}\exp (-(\frac{gMh}{R{T}_{0}}))$$

where ${P}_{0}$ is the standard atmospheric pressure at sea level ($101325{\rm{Pa}}$), *g* is acceleration of gravity (9.8 ${\rm{m}}/{{\rm{s}}}^{2}$), *M* is the dry air molar mass ($0.02896\,Kg/mol$), *R* is the gas constant ($8.31447\,J/(mol\cdot k)$), and *h* is the height above sea level in meters, *T*_0_ is standard temperature at sea level (288.15 K).

The local zenith angle, Z, is defined by

12 $$\cos \,Z=\,\sin \,\phi \,\sin \,\delta +\,\cos \,\phi \,\cos \,\delta \,\cos \,h$$

where $\phi$ is the latitude of the centroid point, and *δ* is the solar declination,

13 $$\begin{array}{c}\delta =0.3723+23.2567\,\sin \,\omega +0.1149\,{\rm{sin2}}\omega -0.1712\,\sin \,3\omega -0.7500\,\cos \,\omega \\ \,\,\,\,\,+\,0.3656\,\cos \,2\omega +0.0201\,\cos \,3\omega \end{array}$$

where $\theta$ is the angle of incidence between the normal slope and the solar beam. A widely used solution for the incidence angle is given by Garnier and Ohmura^46^:

14 $$\cos \,\theta =\,\cos \,\beta \,\cos \,Z+\,\sin \,\beta \,\sin \,Z\,\cos ({\phi }_{sun}-{\phi }_{slop})$$

where *β* is the angle of slope, ${\phi }_{sun}$ and ${\phi }_{slop}$ are the solar azimuth and the azimuth angles of slope.

#### GLUE approach

GLUE, an UA technique, reformulates the model calibration problem as the estimation of posterior probabilities of model response. The GLUE approach has been widely used in the forecasting uncertainty of hydrologic mathematical model^34^. The GLUE approach defines that the performance of simulation is not decided by one specific parameter, but by the combination of parameter sets. We therefore operated the model with a pre-set parameter distribution space, using a random sampling method to obtain a combination of model parameter values, and calculate the likelihood function between the predicted results and observation^47^. The selection of the critical value has certain subjectivity, in this study, the threshold criterion is defined as zero. And parameters with likelihood below this threshold are assigned to zero, that is to say, those parameters do not characterize the model’s functional characteristics in a watershed.
